# Deletion of *Ck2β* gene causes germ cell development arrest and azoospermia in male mice

**DOI:** 10.1111/cpr.12726

**Published:** 2019-11-21

**Authors:** Qiu‐Xia Liang, Zhen‐Bo Wang, Wen‐Long Lei, Fei Lin, Jing‐Yi Qiao, Odile Filhol‐Cochet, Brigitte Boldyreff, Heide Schatten, Qing‐Yuan Sun, Wei‐Ping Qian

**Affiliations:** ^1^ Department of Reproductive Medicine Peking University Shenzhen Hospital Shenzhen Guangdong China; ^2^ State Key Laboratory of Stem Cell and Reproductive Biology Institute of Zoology Chinese Academy of Sciences Beijing China; ^3^ University of Chinese Academy of Sciences Beijing China; ^4^ Center for Reproductive Medicine Nanjing Drum Tower Hospital The Affiliated Hospital of Nanjing University Medical School Nanjing China; ^5^ INSERM U1036 Institute de Recherches en Technologies et Sciences pour le Vivant/Biologie du Cancer et de l'Infection Commissariat à l'Énergie Atomique et aux Énergies Alternatives Grenoble Grenoble France; ^6^ KinaseDetect Krusaa Denmark; ^7^ Department of Veterinary Pathobiology University of Missouri Columbia Missouri

**Keywords:** azoospermia, cell apoptosis, CK2β, spermatogenesis

## Abstract

**Objectives:**

In humans, non‐obstructive azoospermia (NOA) is a major cause of male infertility. However, the aetiology of NOA is largely unknown. Previous studies reported that protein CK2β was abundantly and broadly expressed in spermatogenic cells. Here, we investigate whether protein CK2β participates in spermatogenesis.

**Materials and Methods:**

In this study, we separated spermatogenic cells using STA‐PUT velocity sedimentation, analysed the expression pattern of protein CK2β by immunoblotting, specifically deleted *Ck2β* gene in early‐stage spermatogenic cells by crossing *Ck2β^fl^* mice with *Stra8‐Cre^+^* mice and validated the knockout efficiency by quantitative RT‐PCR and immunoblotting. The phenotypes of *Ck2β^fl/Δ^;SCre^+^* mice were studied by immunohistochemistry and immunofluorescence. The molecular mechanisms of male germ cell development arrest were elucidated by immunoblotting and TUNEL assay.

**Results:**

Ablation of *Ck2β* gene triggered excessive germ cell apoptosis, germ cell development arrest, azoospermia and male infertility. Inactivation of *Ck2β* gene caused distinctly reduced expression of *Ck2α*′ gene and CK2α′ protein.

**Conclusions:**

*Ck2β* is a vital gene for germ cell survival and male fertility in mice.

## 1 INTRODUCTION

Infertility affects about 15% of couples,^1^ and male factor contributes to approximately 50% of all infertility cases in humans.^2^ Azoospermia is a major cause of male infertility, including obstructive azoospermia (OA) and non‐obstructive azoospermia (NOA). NOA, caused by testicular failure, is the most severe form of male infertility. NOA accounts for about 60% of azoospermia and affects about 10% of infertile men.^3^, ^4^ Clinically, NOA can be categorized into hypospermatogenesis, germ cell arrest and Sertoli cell‐only syndrome.^5^ Complex genetic factors contribute to testicular failure, including autosomal chromosome abnormalities,^6^ microdeletions of the Y chromosome^7^, ^8^, ^9^ and single gene mutations. Mutations on dozens of genes, including *SOHLH1*,^10^ *NR5A1*,^11^ *TAF4B*,^12^ *ZMYND15*,^12^ *MCM8*,^13^ *SYCE1*,^14^ *TEX11*,^15^, ^16^ *TDRD7*,^17^ *TDRD9*,^18^ *MEIOB*,^19^ *TEX14*,^19^ *DNAH6*,^19^ *MAGEB4*,^20^ *TEX15*,^21^, ^22^ *FANCM*,^23^, ^24^ *DMC1* 25 and *XRCC2*,^26^ have been identified to cause NOA by pedigree analysis. However, these genes can explain only a part of male infertility, and more related genes need to be explored. Protein kinase CK2, previously known as casein kinase Ⅱ, is a serine/threonine kinase in the form of tetramer with 2 catalytic subunits (α and α') and 2 regulatory β subunits,^27^ functioning in many biological processes like cell proliferation,^28^ apoptosis^29^, ^30^, ^31^ and DNA damage repair.^32^ We have reported that CK2 is vital for oogenesis by participating in apoptosis and DNA damage repair process^33^; CK2β is essential for follicle survival, depletion of which causes massive follicle atresia and eventually premature ovarian failure at young adulthood.^33^

In mice, immunoblotting shows that CK2α, CK2α´ and CK2β are expressed in testis,^34^ and the expression of CK2β is significantly lower than CK2α and CK2α´ in spermatozoa.^35^ Immunohistochemistry reveals that CK2α is localized at the acrosome area of spermatids; CK2α´ appears in the acrosomal and cytoplasmic region of spermatids, whereas CK2β is expressed in spermatogonia and spermatocytes.^35^ Immunofluorescence in spermatozoa reveals that CK2α is localized at the acrosome and mid‐piece; CK2α´ is localized at acrosome, while CK2β displays a weak staining at the acrosome and a strong staining at the mid‐piece.^35^ Ablation of the CK2α' coding gene *Ck2α*′ leads to infertility of male mice, with decreased sperm count and increased spermatozoa with head abnormality.^34^, ^36^ Considering the distinct localization of CK2β and CK2α', it is quite necessary to clarify whether CK2β functions in spermatogenesis and how it functions.

Here, we specifically depleted *Ck2β* gene in spermatogonia by crossing *Ck2β^fl^* mice with *Stra8‐Cre^+^* mice. We found that loss of *Ck2β* gene resulted in germ cell differentiation arrest and male infertility. CK2β is likely to be an essential anti‐apoptotic factor participating in regulating spermatocyte survival.

## 2 MATERIALS AND METHODS

### 2.1 Mice

To obtain *Ck2β^fl/Δ^;SCre^+^* males, we crossed *Stra8‐Cre* 37 with previously reported *Ck2β^fl/fl^* mice,^38^ and the resulting male offspring *Ck2β^Δ/+^;SCre^+^* were mated with *Ck2β^fl/fl^* females to generate *Ck2β^fl/Δ^;SCre^+^* male mice (C57BL/6 and 129/SvEv mixed background). Unless otherwise specified, the *Ck2β^fl/Δ^* male mice were used as the control group. DNA extraction from mouse tail was used to genotype *Ck2β^Δ^*, *Ck2β^fl^* and *Stra8‐Cre* alleles, respectively. The primer pair for *Ck2β^Δ^* allele was forward: 5′‐GAGGGCATAGTAGATATGAATCTG‐3′ and reverse: 5′‐GGATAGCAAACTCTCTGAG‐3′. The primer pair for *Ck2β^fl^* allele was forward: 5′‐ATGAGTAGCTCTGAGGAGGTG‐3′ and reverse: 5′‐GGATAGCAAACTCTCTGAG‐3′. The primer pair for *Stra8‐Cre* allele was forward: 5′‐GTGCAAGCTGAACAACAGGA‐3′ and reverse: 5′‐AGGGACACAGCATTGGAGTC‐3′. All animal operations were carried out in accordance with the guidelines of the Animal Research Committee principles of the Institute of Zoology, Chinese Academy of Sciences. All mice were housed in a temperature‐controlled room with a 12D:12L cycle.

### 2.2 Antibodies

Antibodies used in the experiments were obtained from the following companies: rabbit monoclonal anti‐CK2β antibody (1:1000) (ABGENT; AJ1128b); mouse monoclonal anti‐β‐ACTIN antibody (1:1000) (Easybio Technology; BE0021); mouse monoclonal anti‐MVH antibody (1:200) (Abcam; ab27591); mouse monoclonal anti‐CK2α antibody (1:1000) (Abcam; ab70774); and rabbit polyclonal anti‐CK2α′ antibody (1:1000) (Bioworld Technology; BS6571); secondary antibodies were purchased from Zhongshan Golden Bridge Biotechnology Co, LTD.

### 2.3 Isolation of mouse spermatogenic cells

Considering the sequential and synchronized occurrence of Sertoli cells, spermatogonia, spermatocytes and round spermatids in the postnatal mouse testis, isolation was performed from samples at 6, 8 and 17 days postpartum (dpp) and adult mice, respectively.^39^ The isolation procedures were described previously.^39^, ^40^ Briefly, testes from *Ck2β^fl/Δ^* and *Ck2β^fl/Δ^;SCre^+^* mice were dissected and decapsulated. The seminiferous tubules were minced into pieces and incubated in 8 mL phosphate‐buffered saline (PBS) containing 100 μL 1 mg/mL collagenase (Sigma, C5138) and 100 μL 1 mg/mL hyaluronidase (Sigma, H3506) at 37℃ for 15 minutes with gentle shaking. After centrifugation at 4℃, 200 g for 5 minutes, the cells were collected, washed with PBS, incubated in 15 mL PBS with 0.25% trypsin (Gibco, 25200‐072) and 1 mg/mL DNase I (AppliChem; A3778,0050) at 37℃ for 15 minutes with gentle shaking. After filtration using a 40‐μm nylon cell strainer and sedimentation for 3 hours, the cells were separated by 2%‐4% bovine serum albumin (BSA) gradient in PBS. The cell pools (10 mL/pool) were collected separately in numbered tubes and centrifuged at 4℃, 600 g for 5 minutes, and then the supernatant was removed. Adding 1 mL PBS to each odd‐numbered tube, the cells were resuspended, and then, 60 µL suspension of each tube was added to 96‐cell plate for further observation. The cellular purity and cell types were identified under phase contrast microscope based on morphological evaluation and the diameter of cells.^39^ Expected cell types with cellular purity (≥90%) were collected for immunoblotting.

### 2.4 Immunoblotting

The tunica albuginea of testes was removed, the seminiferous tubules were homogenized using a homogenizer in RIPA buffer (25 mM Tris–HCl, pH 7.6, 350 mM NaCl, 1% Nonidet P‐40, 1% sodium deoxycholate and 0.1% sodium dodecyl sulphate) (Solarbio Life Sciences; R0010) supplemented with protease and phosphatase inhibitor cocktail (Roche Diagnostics, 04693116001). Spermatogenic cells isolated from testes were resuspended in the above‐described RIPA buffer. After transient sonication, the lysates were incubated on ice for 30 minutes and then centrifuged at 4℃, 14 000 g for 20 minutes. The supernatant was transferred to a new tube, and equal volume loading buffer was added. After being boiled at 95℃ for 10 minutes, the protein lysates were used for immunoblotting analysis. Immunoblotting was performed as described previously.^41^ Briefly, the separated proteins in SDS‐PAGE were electrically transferred to a polyvinylidene fluoride membrane. After incubation with primary and secondary antibodies, the membranes were scanned with Bio‐Rad ChemiDoc XRS+.

### 2.5 Quantitative RT‐PCR

Total RNA of testes from *Ck2β^fl/Δ^* and *Ck2β^fl/Δ^;SCre^+^* male mice was extracted using the RNeasy Micro Kit (Qiagen, 74004), and the first‐strand cDNA was generated with cDNA synthesis kit (Invitrogen; 11754050). *Glyceraldehyde‐3‐phosphate dehydrogenase (Gapdh)* or *β‐actin* was used as internal control to normalize the cDNA level of the samples. The experiment was conducted by using UltraSYBR Mixture (CoWin Biosciences; CW0957) in Roche LightCycler480 II detection system. The relative gene expression was calculated by the 2^−ΔΔCt^ method. The primers used were as follows.

*Ck2α* forward: 5′‐CTTCGGCTAATAGACTGGGGT‐3′;
*Ck2α* reverse: 5′‐TCGAGAAGCAACTCGGACATT‐3′.
*Ck2α*′ forward: 5′‐TCCCGAGCTGGGGTAATCAA‐3′;
*Ck2α*′ reverse: 5′‐TGTTCCACCACGAAGGTTCTC‐3′.
*Ck2β* forward: 5′‐AATGAGCAGGTGCCTCACTAT‐3′;
*Ck2β* reverse: 5′‐TGTTCGATCAAGTCGCTCTGG‐3′.
*Gapdh* forward: 5′‐CCCCAATGTGTCCGTCGTG‐3′;
*Gapdh reverse:* 5′‐TGCCTGCTTCACCACCTTCT‐3′.
*β‐Actin* forward: 5′‐GGCTGTATTCCCCTCCATCG‐3′;
*β‐Actin* reverse: 5′‐CCAGTTGGTAACAATGCCATGT‐3′.

### 2.6 Tissue collection and histological analysis and immunofluorescence

Testes or caudal epididymides were dissected from *Ck2β^fl/Δ^* and *Ck2β^fl/Δ^;SCre^+^* male mice immediately after euthanasia. Testes were fixed in 4% paraformaldehyde (pH 7.4) or Bouin's fixative overnight at 4℃. Caudal epididymides were fixed in 4% paraformaldehyde (pH 7.4) overnight at 4℃. The tissues were then dehydrated in a graded ethanol series, cleaned in xylene and embedded in paraffin. The paraffin‐embedded tissues were sectioned into 5 μm and mounted on glass slides. After adequately drying at 48℃, the sections were deparaffinized in xylene, hydrated in a graded ethanol series and stained with haematoxylin and eosin for histological analysis.

Testes for immunofluorescent staining were fixed in 4% paraformaldehyde (pH 7.4) overnight at 4℃, dehydrated and embedded in paraffin. The paraffin‐embedded testes were sectioned into 5 μm and mounted on glass slides. The sections were then deparaffinized in xylene, hydrated in a graded ethanol series, immersed in sodium citrate buffer (pH 6.0) and heated for 15 minutes in a microwave oven for antigen retrieval. After blocking with 5% donkey serum albumin, the sections were incubated with primary antibody at 4℃ overnight and appropriate TRITC‐conjugated secondary antibody. The nuclei were stained with 4',6‐diamidino‐2‐phenylindole (DAPI). Images were captured using a laser scanning confocal microscope (Zeiss 780 META).

### 2.7 TUNEL assay

TUNEL assay was performed in accordance with the DeadEnd^TM^ Fluorometric TUNEL System (Promega Biosciences, G3250). Images were captured using a laser scanning confocal microscope (Zeiss 780 META).

### 2.8 Breeding assay

C57BL/6J wild‐type female mice with known fertility were mated with 8‐week‐old *Ck2β^fl/Δ^* and *Ck2β^fl/Δ^;SCre^+^* male mice. For 6 months, the cages were monitored daily to record the number of pups and litter sizes.

### 2.9 Statistical analysis

Paired two‐tailed Student's *t* test was used for statistical analysis. The data were considered statistically significant when *P* < .05 (*), .01 (**) or .001 (***).

## 3 RESULTS

### 3.1 Deletion of *Ck2β* by *Stra8‐Cre* results in male infertility

**Figure 1:**
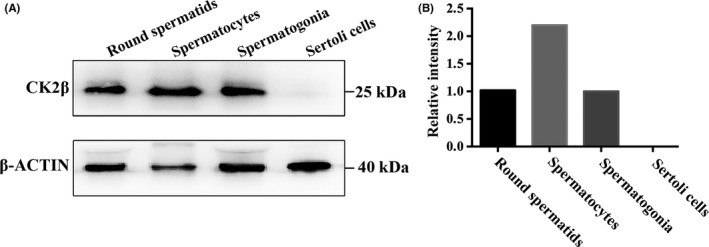
The expression of CK2β protein in different types of cells in mouse testis. A, Immunoblotting detection of the expression pattern of CK2β protein in mouse round spermatids, spermatocytes, spermatogonia and Sertoli cells. β‐Actin was detected as an internal control. Molecular mass is given in kilo Daltons. B, Relative intensity of protein CK2β in mouse round spermatids, spermatocytes, spermatogonia and Sertoli cells. The immunoblotting results were analysed using imageJ

To study the potential role of protein CK2β during spermatogenesis, we first isolated the spermatogonia, spermatocytes, round spermatids and Sertoli cells from mouse testes to detect the expression of CK2β. Immunoblotting analysis showed that CK2β was expressed from spermatogonia to round spermatids and the highest expression level was detected in spermatocytes, while no expression was detected in Sertoli cells (Figure 1A and 1B). These results suggest that CK2β may participate in spermatogenesis.

**Figure 2:**
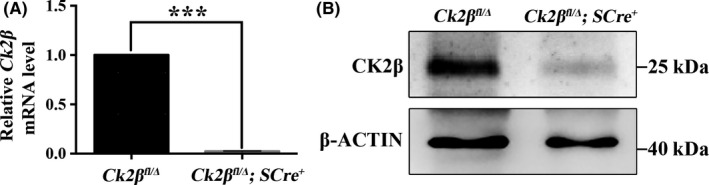
Verification of *Ck2β* deletion in mouse testis. A, Quantitative RT‐PCR showing the expression of *Ck2β* in testes from *Ck2β^fl/Δ^* and *Ck2β^fl/Δ^;SCre^+^* mice. *Gapdh* was detected as an internal control. Experiment was repeated at least 3 times. Data are presented as the mean ± SEM *P* < .001 (***). B, Immunoblotting detecting the expression of protein CK2β in *Ck2β^fl/Δ^* and *Ck2β^fl/Δ^;SCre^+^* testes. Level of β‐ACTIN was used as internal control. Experiment was repeated at least 3 times. Molecular mass is given in kilo Daltons

To explore the potential function of protein CK2β during spermatogenesis, we mated *Ck2β^fl^* mice, in which exons I–II were targeted, with *Stra8‐Cre^+^* mice to generate *Ck2β^fl/Δ^;Stra8‐Cre^+^* mice (referred to as *Ck2β^fl/Δ^;SCre^+^*)(Figure S1). In *Stra8‐Cre^+^* mice, Cre recombinase was specifically expressed in early‐stage spermatogonia from 3 days after birth onward and peaked in preleptotene spermatocytes at 7 dpp.^37^ Quantitative RT‐PCR and immunoblotting analysis confirmed that the expression of CK2β in testes from *Ck2β^fl/Δ^;SCre^+^* mice was efficiently deleted at both mRNA and protein levels (Figure 2A, 2B and Figure S2).

To determine the effects of *Ck2β* depletion on male fertility, we carried out a breeding assay by mating *Ck2β^fl/Δ^* or *Ck2β^fl/Δ^;SCre^+^* male mice with C57BL/6J wild‐type females of tested fertility for 6 months. Continuous breeding observation indicated that *Ck2β^fl/Δ^;SCre^+^* males were completely infertile (Table 1).

**Table 1:** Statistical analysis of average litter size and pups per litter of *Ck2β^fl/Δ^* and *Ck2β^fl/Δ^;SCre^+^* males

Breeding assay of *Ck2β^fl/Δ^; SCre^+^* males in 6 mo			
Group	Number	Litters/male (mean ± SEM)	Pups/litter (mean ± SEM)
*Ck2β^fl/Δ^*	6	7.67 ± 0.75	8.11 ± 1.45
*Ck2β^fl/Δ^*; *SCre^+^*	6	0	0

### 3.2 *Ck2β* depletion causes testicular atrophy and azoospermia

**Figure 3:**
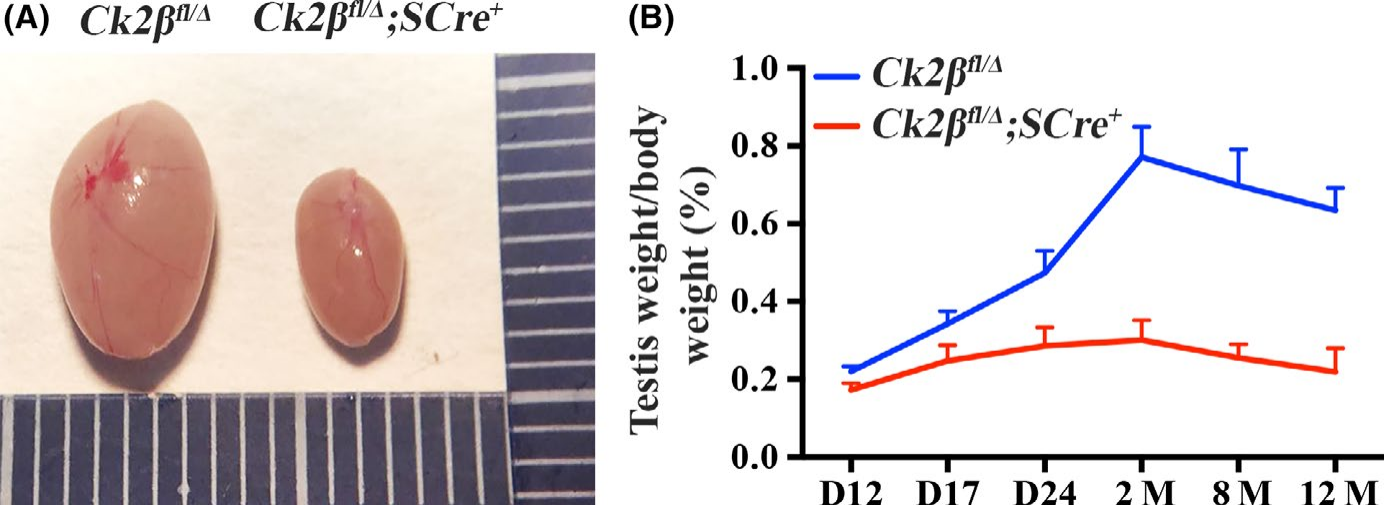
*Ck2β* knockout in mouse germ cells by *Stra8‐Cre* resulted in testicular atrophy. A, Representative image of testes from *Ck2β^fl/Δ^* and *Ck2β^fl/Δ^;SCre^+^* mice at 2 months of age. B, Testis weight‐to‐body weight ratio of *Ck2β^fl/Δ^* and *Ck2β^fl/Δ^;SCre^+^* mice of days 12, 17, 24, months 2, 8 and 12 after birth. For each time point, at least 3 mice of each genotype were used for the analysis. Data are presented as the mean ± SEM

To determine the causes of infertility in *Ck2β^fl/Δ^;SCre^+^* males, we first examined whether the infertility was due to testicular dysfunction and the consequential functional azoospermia. *Ck2β^fl/Δ^;SCre^+^* males were observed to develop normally, without defects until at least 12 months of age. However, the testes of adult *Ck2β^fl/Δ^;SCre^+^* males exhibited a marked reduction in size (Figure 3A). Statistical analysis revealed that the testis weight ratio of the testes of *Ck2β^fl/Δ^* continued to increase from day 12 to month 2 after birth and slightly decreased from month 2 to month 12, with a mean testis weight ratio of 0.2493%, 0.3088%, 0.4736%, 0.7709%, 0.6978% and 0.6345% corresponding to days 12, 17, 24, months 2, 8 and 12 after birth, respectively (Figure 3B). By comparison, the testis weight ratio of *Ck2β^fl/Δ^;SCre^+^* males showed small changes from day 12 to month 12 after birth, with a mean testis weight ratio of 0.1942%, 0.2454%, 0.2859%, 0.3012%, 0.2550% and 0.2194% corresponding to days 12, 17, 24, months 2, 8 and 12 after birth, respectively (Figure 3B).

**Figure 4:**
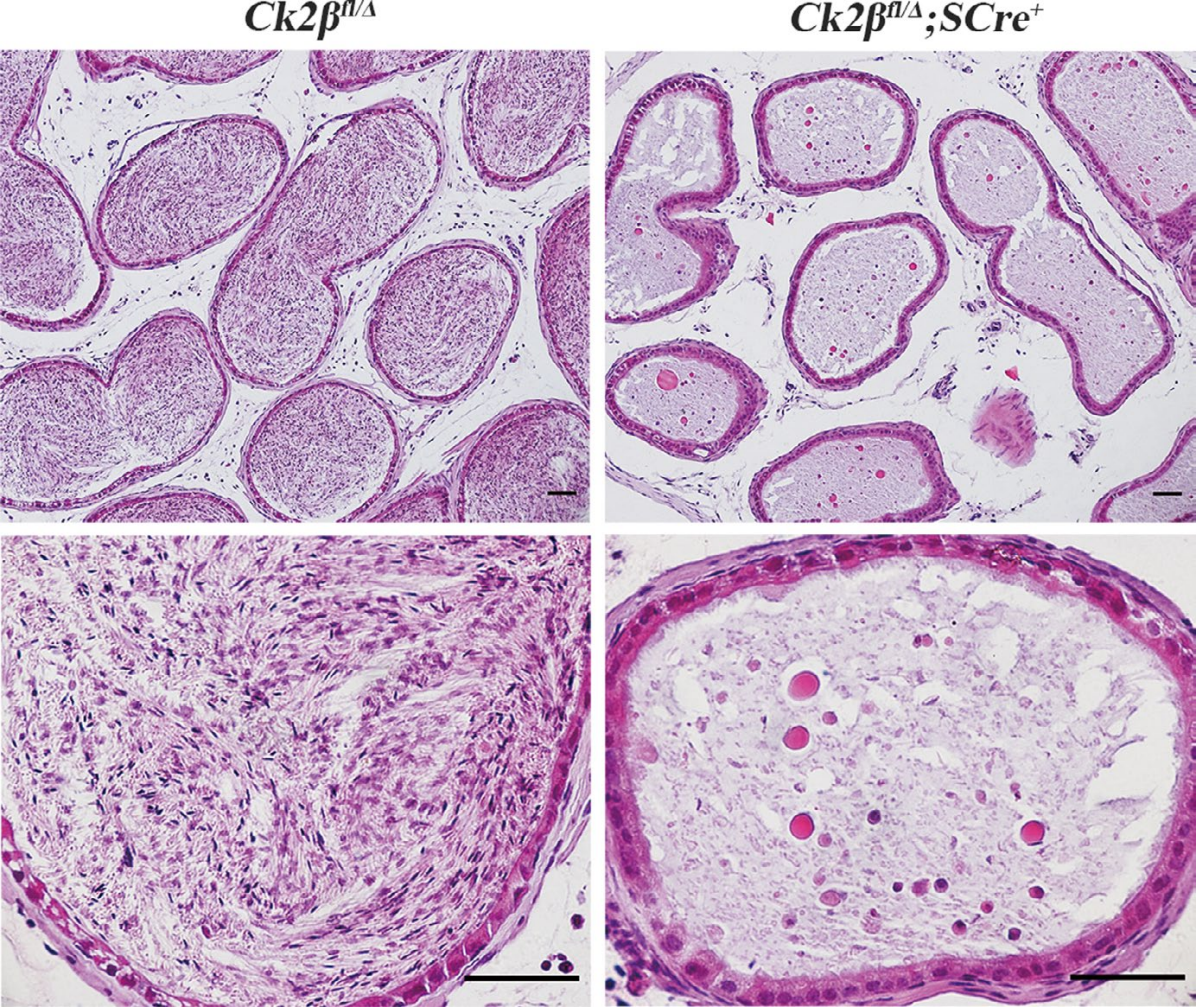
Histological analysis of the caudal epididymides from *Ck2β^fl/Δ^* and *Ck2β^fl/Δ^;SCre^+^* mice at 2 mo of age. Scale bars, 50 μm, upper panel; 50 μm, lower panel

Further histological analysis showed that no mature spermatozoa were present in the epididymal lumens of *Ck2β^fl/Δ^;SCre^+^* males (Figure 4). These data demonstrate that *Ck2β* deletion results in testicular atrophy and azoospermia in mice, and these phenotypes do not change with increasing age after adulthood.

### 3.3 *Ck2β* knockout results in spermatogenesis arrest in male mice

To clarify the cause of testicular atrophy and azoospermia in *Ck2β^fl/Δ^;SCre^+^* males, we next examined the first wave of spermatogenesis at 12 dpp, 15 dpp and 17 dpp corresponding to leptotene, zygotene and pachytene spermatocytes, respectively.

**Figure 5:**
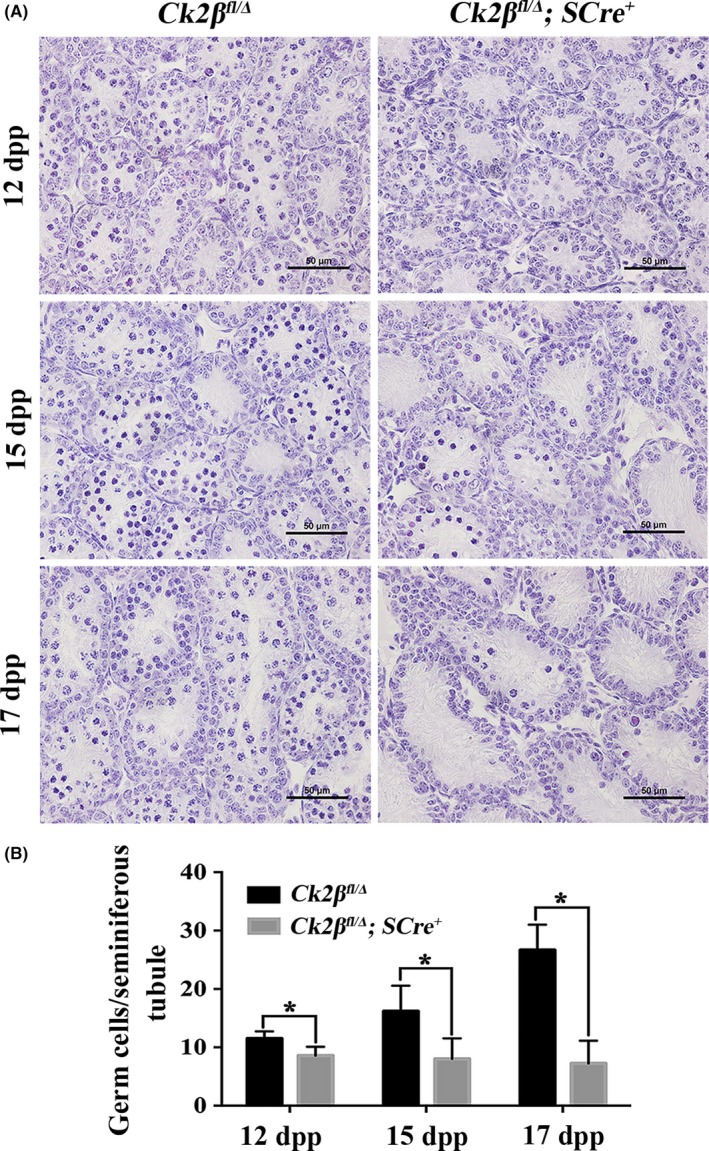
Reduced germ cells in testes from juvenile *Ck2β^fl/Δ^;SCre^+^* mice. A, Histological analysis of the seminiferous tubules of *Ck2β^fl/Δ^* and *Ck2β^fl/Δ^;SCre^+^* mice at 12, 15 and 17 dpp (days postpartum). Scale bars, 50 μm. B, Quantification of the germ cells in the seminiferous tubules of *Ck2β^fl/Δ^* and *Ck2β^fl/Δ^;SCre^+^* mice at 12, 15 and 17 dpp. For each time point, at least 100 tubules from 3 mice of each genotype were used for the analysis. Data are presented as the mean ± SEM *P* < .05 (*)

As shown in Figure 5A, the testes of *Ck2β^fl/Δ^* males were populated by spermatocytes of various stages, while the seminiferous tubules of *Ck2β^fl/Δ^;SCre^+^* mice contained only few spermatocytes. Statistical analysis revealed that the number of germ cells of testes from *Ck2β^fl/Δ^* males continued to rise from 12 dpp to 17 dpp, but these numbers were almost unchanged in *Ck2β^fl/Δ^;SCre^+^* males (Figure 5B), suggesting that *Ck2β* knockout affected the prophase of meiosis I at the first wave of spermatogenesis in mice.

**Figure 6:**
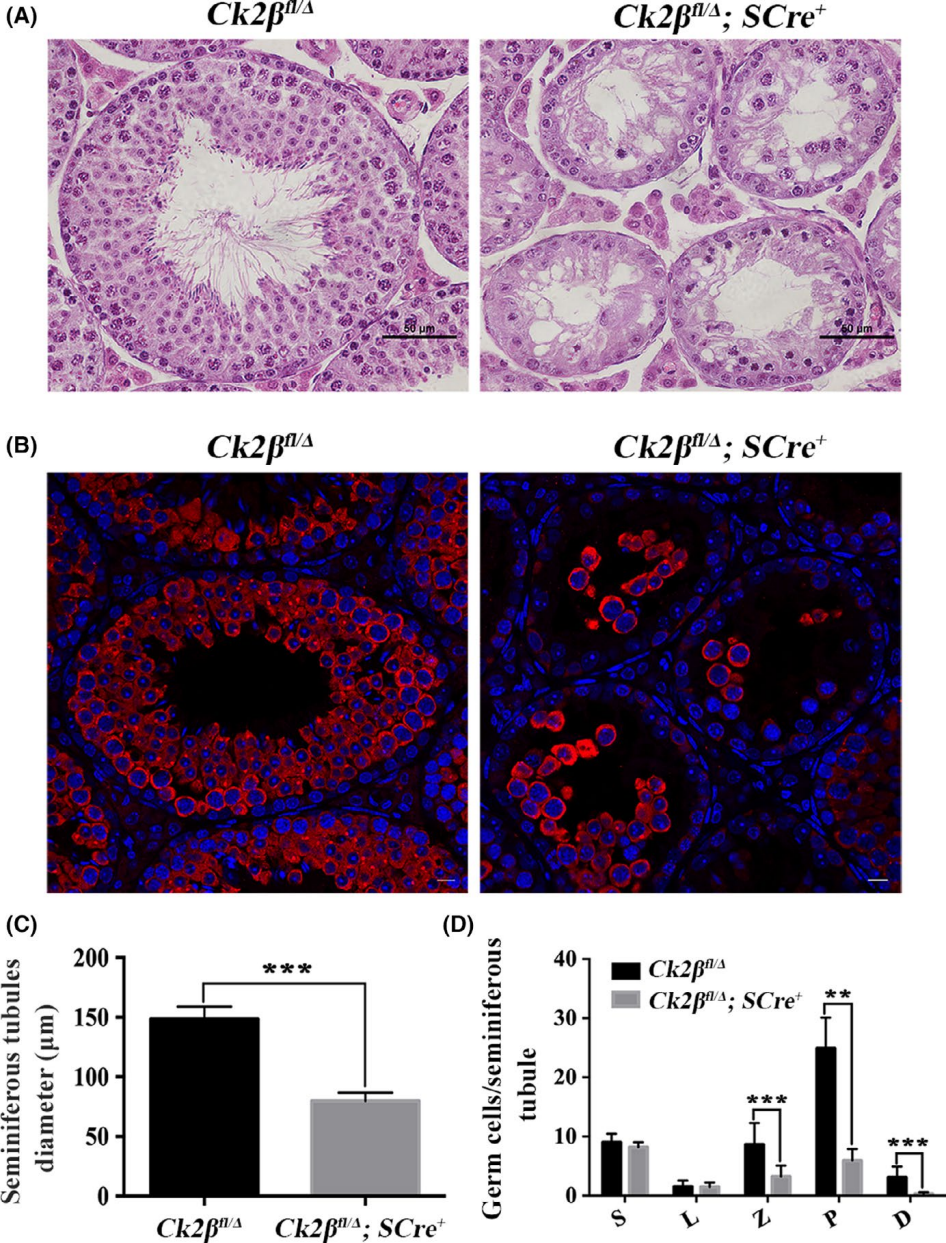
Massive germ cell loss in the testes from *Ck2β^fl/Δ^;SCre^+^* mice at 2 mo of age. A, Histological analysis of the seminiferous tubules of *Ck2β^fl/Δ^* and *Ck2β^fl/Δ^;SCre^+^* mice at 2 mo of age. Scale bars, 50 μm. B, Immunofluorescence detection of MVH‐positive germ cells in the seminiferous tubules from *Ck2β^fl/Δ^;SCre^+^* mice at 2 mo of age. Germ cells were labelled with anti‐MVH antibody (red) and DAPI (blue). Scale bars, 10 μm. C, The diameter of the seminiferous tubules in *Ck2β^fl/Δ^* and *Ck2β^fl/Δ^;SCre^+^* mice. At least 60 tubules from 3 mice of each genotype were used for the analysis. Data are presented as the mean ± SEM *P* < .001 (***). D, Quantification of the germ cells in the seminiferous tubules of *Ck2β^fl/Δ^* and *Ck2β^fl/Δ^;SCre^+^* mice at 2 mo of age. S, spermatogonia; L, leptotene spermatocytes; Z, zygotene spermatocytes; P, pachytene spermatocytes; D, diplotene spermatocytes. At least 60 tubules from 5 mice of each genotype were used for the analysis. Data are presented as the mean ± SEM *P* < .01 (**), *P* < .001 (***)

To determine whether this effect would continue to adulthood, we examined the seminiferous tubules of both *Ck2β^fl/Δ^* and *Ck2β^fl/Δ^;SCre^+^* males at 2 months of age. Histological analysis showed that the seminiferous tubules of *Ck2β^fl/Δ^* males were filled with spermatogonia, spermatocytes, round or elongated spermatids (Figure 6A); however, there are only a few germ cells and no round or elongated spermatids in the seminiferous tubules of the *Ck2β^fl/Δ^;SCre^+^* males (Figure 6A). To confirm these observations, we performed immunofluorescence of the germ cell marker MVH on testicular sections. The results showed that the number of MVH‐positive germ cells was dramatically reduced in *Ck2β^fl/Δ^;SCre^+^* tubules compared with those in *Ck2β^fl/Δ^* tubules (Figure 6B). The diameter of the seminiferous tubules of *Ck2β^fl/Δ^;SCre^+^* males dropped to half of that in *Ck2β^fl/Δ^* males (Figure 6C).

To precisely explore the stage affected by *Ck2β* knockout during spermatogenesis, we analysed the numbers of spermatogonia, leptotene, zygotene, pachytene and diplotene spermatocytes in *Ck2β^fl/Δ^* and *Ck2β^fl/Δ^;SCre^+^* males at 2 months of age. Germ cell types were identified based on the size, the positioning in seminiferous tubules, the status of chromatin (the amount and distribution of heterochromatin, chromatin threads thickness and looseness) of cells.^42^ The results demonstrated that the numbers of zygotene, pachytene and diplotene spermatocytes were significantly reduced, but that of spermatogonia and leptotene spermatocytes were unchanged in *Ck2β^fl/Δ^;SCre^+^* males, compared with *Ck2β^fl/Δ^* males. These findings suggest that *Ck2β* knockout may not affect spermatogonial proliferation, differentiation and entrance into meiosis in mice, but severely impairs spermatocyte survival and causes spermatogenesis arrest (Figure 6D).

### 3.4 Deletion of *Ck2β* triggers germ cell apoptosis

**Figure 7:**
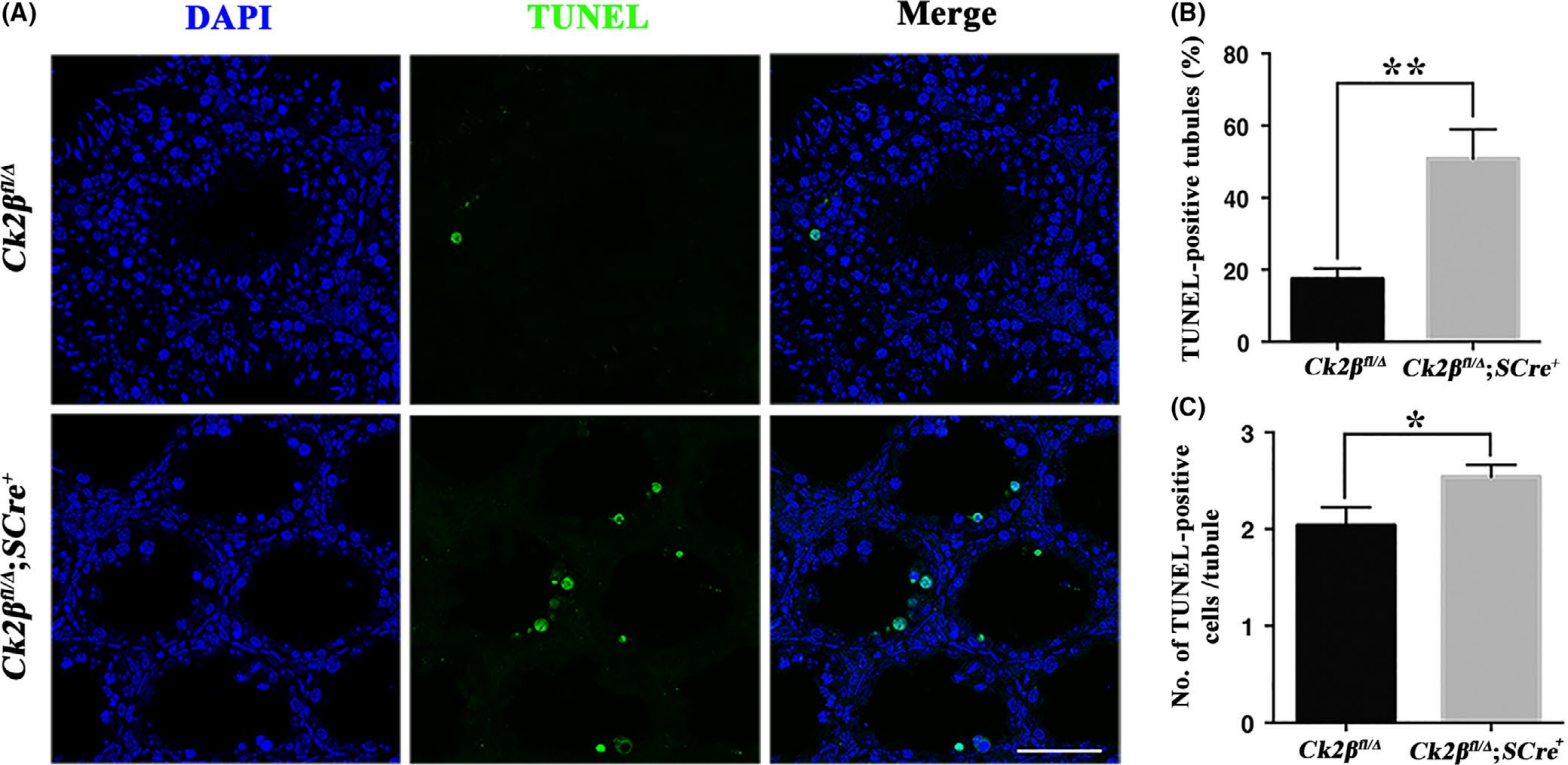
Apoptosis detection in *Ck2β^fl/Δ^* and *Ck2β^fl/Δ^;SCre^+^* testes. A, Representative TUNEL assay patterns in testes from *Ck2β^fl/Δ^* and *Ck2β^fl/Δ^;SCre^+^* mice at 2 mo of age. TUNEL‐positive germ cells: green. Nuclei were stained with DAPI (blue). Scale bars, 50 μm. B, Quantification of TUNEL‐positive seminiferous tubules. At least 200 tubules from 3 mice of each genotype were used for the analysis. Data are presented as the mean ± SEM *P* < .01 (**). C, Number of TUNEL‐positive germ cells per tubule. At least 200 tubules from 3 mice of each genotype were used for the analysis. Data are presented as the mean ± SEM *P* < .05 (*)

To further examine the causes of decreased numbers of spermatocytes, detection of apoptosis in testes was carried out by the TUNEL assay. The results showed that more than half of the tubules had TUNEL‐positive germ cells in *Ck2β^fl/Δ^;SCre^+^* males compared with less than 20% of that in *Ck2β^fl/Δ^* males (Figure 7A and 7B). Besides, the number of TUNEL‐positive germ cells was also obviously increased in *Ck2β^fl/Δ^;SCre^+^* males (Figure 7A and 7C). The above data indicated that deletion of *Ck2β* triggered germ cell apoptosis, and consequently caused reduction in the number of spermatocytes.

### 3.5 Inactivation of *Ck2β* causes distinctly reduced expression of Ck2α′ but not Ck2α

**Figure 8:**
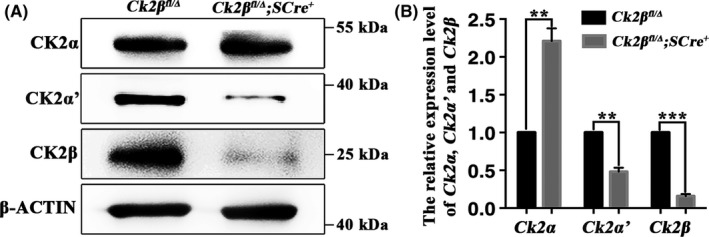
Detection of the expression of CK2α, CK2α’ and CK2β in testes from *Ck2β^fl/Δ^* and *Ck2β^fl/Δ^;SCre^+^* mice at protein and mRNA level. A, Immunoblotting detection of the expression of CK2α, CK2α’ and CK2β in testes from *Ck2β^fl/Δ^* and *Ck2β^fl/Δ^;SCre^+^* mice at 2 mo of age. β‐Actin was detected as an internal control. Molecular mass is given in kilo Daltons. Experiment was repeated at least 3 times. B, Quantitative RT‐PCR showing the expression of *Ck2α*, *Ck2α’* and *Ck2β* in testes from *Ck2β^fl/Δ^* and *Ck2β^fl/Δ^;SCre^+^* mice at 2 mo of age. *β‐Actin* was detected as an internal control. Experiment was repeated at least 3 times. Data are presented as the mean ± SEM *P* < .01 (**), *P* < .001 (***)

To clarify the forms of CK2 functions in spermatogenesis, immunoblotting was carried out to assess protein levels of CK2α, CK2α' and CK2β in testes of *Ck2β^fl/Δ^* and *Ck2β^fl/Δ^;SCre^+^* mice. Compared with *Ck2β^fl/Δ^* mice, the level of CK2β protein, as expected, was significantly reduced in testis extracts from *Ck2β^fl/Δ^;SCre^+^* mice (Figure 8A and Figure S3). Meanwhile, the level of CK2α' protein was also significantly down‐regulated in testes of *Ck2β^fl/Δ^;SCre^+^* mice (Figure 8A and Figure S3). By comparison, the levels of CK2α protein showed no variation in testes of *Ck2β^fl/Δ^* and *Ck2β^fl/Δ^;SCre^+^* mice (Figure 8A and Figure S3). The results suggest that CK2 presumably functions in the forms of α'_2_β_2_ in spermatogenesis and the reduced expression of CK2α' protein in testes of *Ck2β^fl/Δ^;SCre^+^* is assumably due to degradation resulting from decreased stability of CK2α' protein without CK2β.

To test the hypothesis, the expression of *Ck2α*, *Ck2α*′ and *Ck2β* in the mRNA level was detected in testes by RT‐PCR. Results found that the mRNA expression of both *Ck2α*′ and *Ck2β* in testes of *Ck2β^fl/Δ^* was significantly higher than that in *Ck2β^fl/Δ^;SCre^+^* (Figure 8B and Figure S4), while the mRNA expression of *Ck2α* in testes of *Ck2β^fl/Δ^* was remarkably lower than it in testes of *Ck2β^fl/Δ^;SCre^+^* (Figure 8B and Figure S4).

## 4 DISCUSSION

NOA is problematic for males as it results in infertility. Accumulating studies have shown that gene mutations contribute to NOA; however, the molecular mechanisms underlying the disorder are still poorly understood. Immunoblotting revealed that protein CK2β was expressed in spermatogonia, spermatocytes and round spermatids, and no expression was seen in Sertoli cells. These findings are consistent with previous reports that CK2β protein is abundantly and broadly expressed in early spermatogenesis as shown by in situ hybridization and immunohistochemistry in mice.^34^, ^35^ However, our results are inconsistent with researches on rats,^43^ which indicates that CK2β protein is located in both germ cells and Sertoli cells. This discrepancy may be caused by variations between species.

A study reports that *Ck2α^‐/‐^* mice die in mid‐embryogenesis with severe developmental defects in the neural tube and heart,^44^ but there is no specific study of its role in gametogenesis. The fertility of *Ck2α*′*^‐/‐^* females is not affected, while *Ck2α*′*^‐/‐^* males are infertile.^34^ Male mice lacking *Ck2α*′ displayed oligozoospermia and globozoospermia; germ cells display extensive degenerative changes characterized by nuclear abnormalities at the stages from spermatogonia to early spermatids, including the first spermatogenesis wave.^34^, ^36^ Zygote‐specific knockout of *Ck2β* results in embryonic lethality after implantation with decreased cell proliferation but no signs of apoptosis.^38^ We have reported that oocyte‐specific knockout of *Ck2β* given rise to ovarian follicle atresia and POF, which are related to down‐regulated PI3K/AKT signalling and failed DNA damage response signalling.^33^

In this study, using *Stra8* promoter‐driven Cre recombinase, *Ck2β* gene is effectively deleted in male mouse germ cells from the early stage of spermatogonia, which facilitated to explore the roles of *Ck2β* in spermatogenesis. The weak expression of protein CK2β present in the testes of *Ck2β^fl/Δ^;SCre^+^* mice probably comes from Leydig cells or other somatic cells in testes. In consistence with the result of *Ck2α*′ knockout,^34^ *Ck2β* mutant male mice are also infertile. *Ck2β* deletion severely blocks the first wave of spermatogenesis. The number of spermatogonia and leptotene spermatocytes has no change in adult mice, while the number of zygotene, pachytene and diplotene spermatocytes is significantly lower in *Ck2β* mutant mice than control mice. It seems that CK2β protein functions at the spermatocyte stage but not spermatogonia stage or the onset of meiotic prophase. Furthermore, the increased number of apoptotic spermatocytes implies that CK2β protein plays a critical role in spermatocyte survival. However, it is unknown whether CK2β protein functions in spermiogenesis because the blockage of spermatogenesis by *Ck2β* deletion occurs at the spermatocyte stage. Considering studies in somatic cells^29^, ^30^, ^31^, ^32^ and oocytes,^33^ it is likely that CK2β protein functions via apoptosis or DNA damage signalling pathways in mouse spermatogenesis.

In this study, we find that deletion of protein CK2β in testes results in significant reduction in protein CK2α′. In contrast, the level of protein CK2α shows no obvious difference in *Ck2β* mutant testes and control testes. Considering previous reports that CK2α′ protein is indispensable to spermatogenesis,^34^ CK2 presumably functions in spermatogenesis in the form of α′_2_β_2_, and the degradation of CK2α′ protein is caused by decreased stability of CK2α' protein without CK2β. However, down‐regulation of *Ck2α*′ at the mRNA level is observed in *Ck2β* mutant, indicating that *Ck2β* knockout disrupts *Ck2α*′ transcription and consequently disturbs the function of CK2α′ protein per se or as a catalytic subunit of CK2 holoenzyme. In addition, it is confusing that CK2α has no changes in the protein level while the mRNA expression is obviously elevated. From above results, the mutual regulation of the three subunits is complicated.

In summary, we identify *Ck2β* as a vital gene for germ cell survival and male fertility in mice, but whether it is a candidate gene for NOA in humans needs further clarification.

## CONFLICT OF INTEREST

The authors declare no conflict of interest.

## AUTHOR CONTRIBUTIONS

QXL, ZBW, FL, QYS and WPQ designed the experiments; QXL, FL, LWL and JYQ performed experiments and analysed data; QXL wrote the manuscript; QXL, QYS, OFC, BB and HS revised manuscript. All authors read and approved the final revised manuscript.

## Data Availability

The data that support the findings of this study are available from the corresponding author upon reasonable request.
