# Supplementary figures and images for: Deletion of Ck2β gene causes germ cell development arrest and azoospermia in male mice
Source: Cell Prolif. 2019 Nov 21;53(1):e12726. doi: 10.1111/cpr.12726 (PMC6985669; doi:10.1111/cpr.12726)

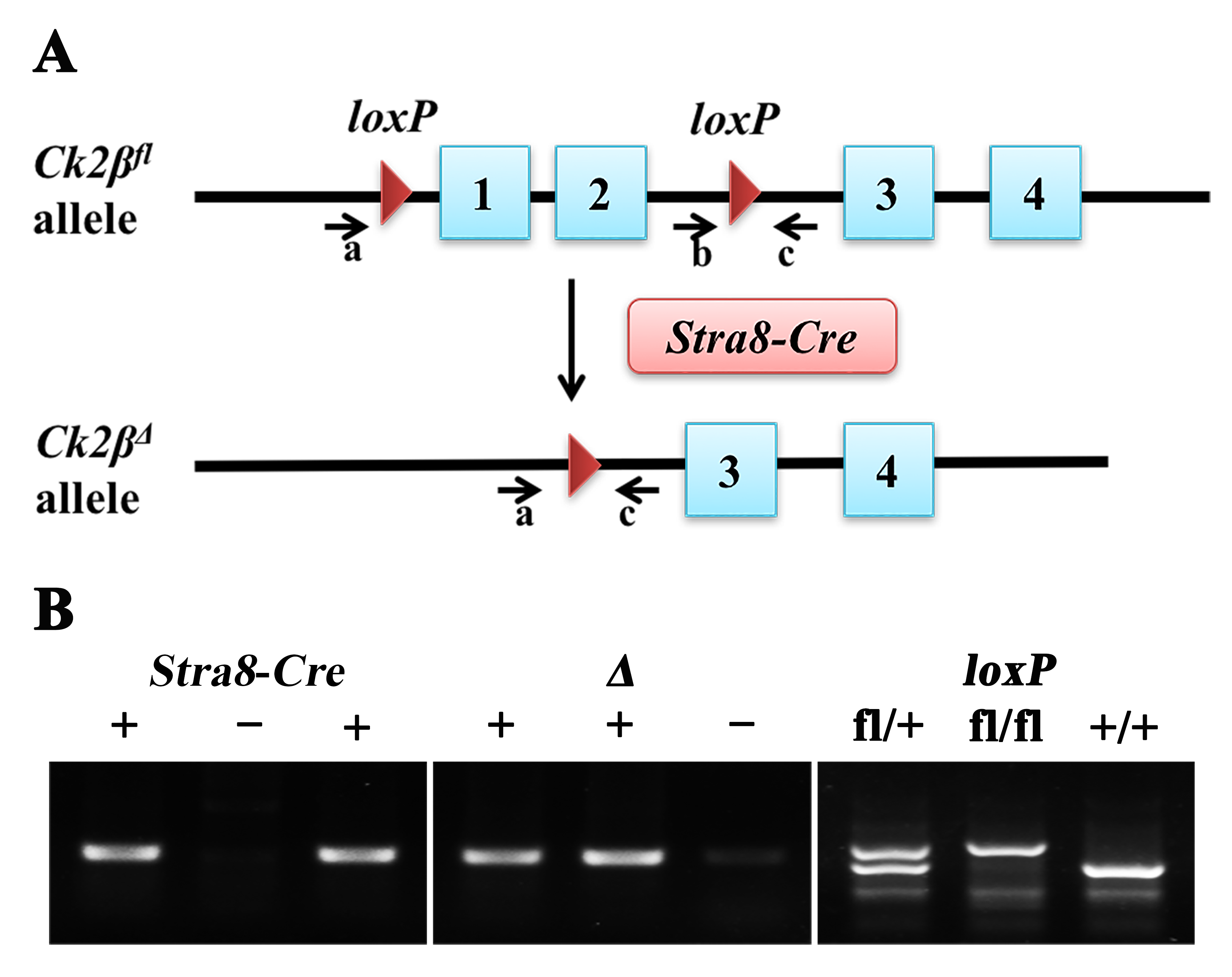

Supplement: Supplementary file 1 [file CPR-53-e12726-s001.png]

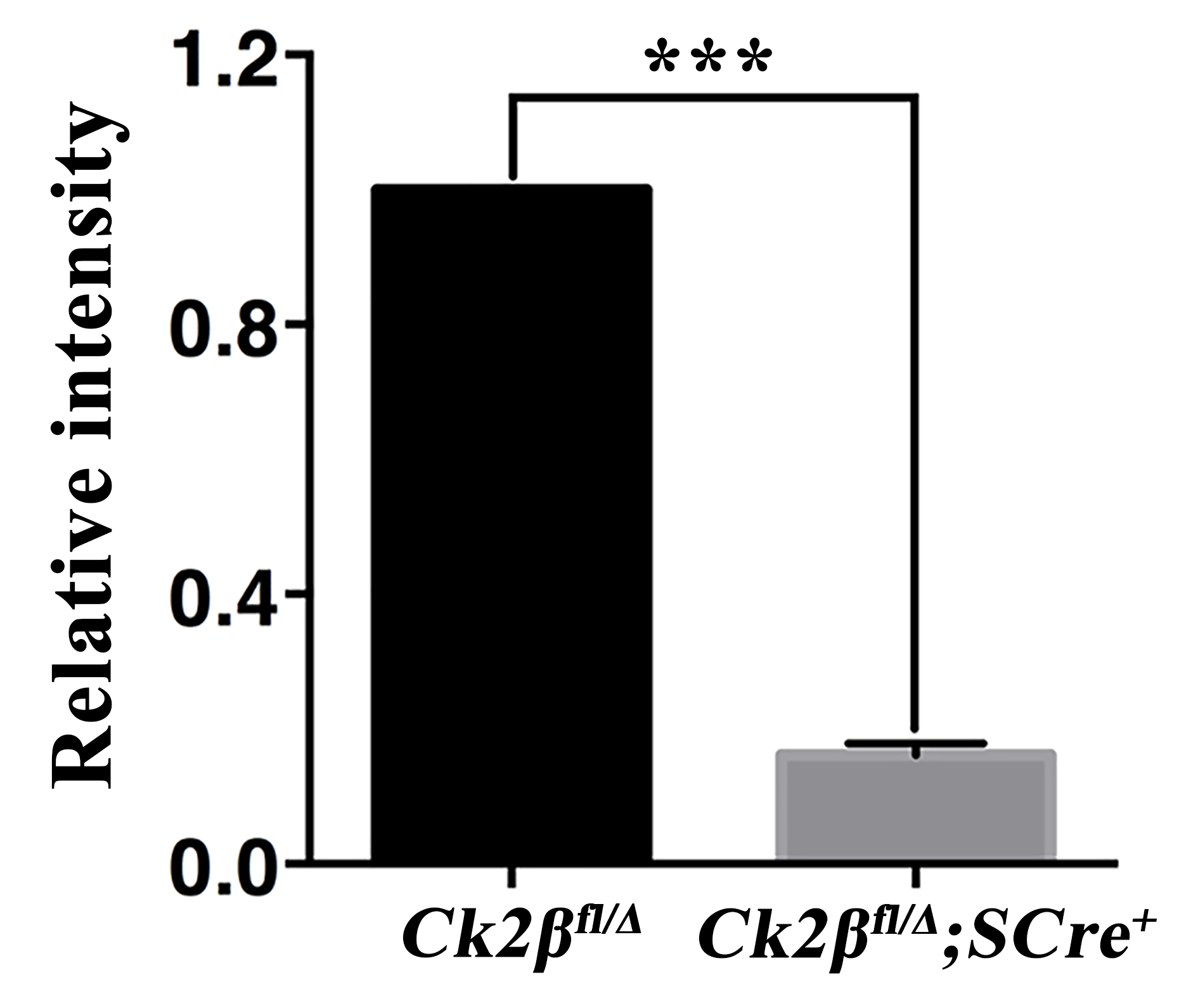

Supplement: Supplementary file 2 [file CPR-53-e12726-s002.png]

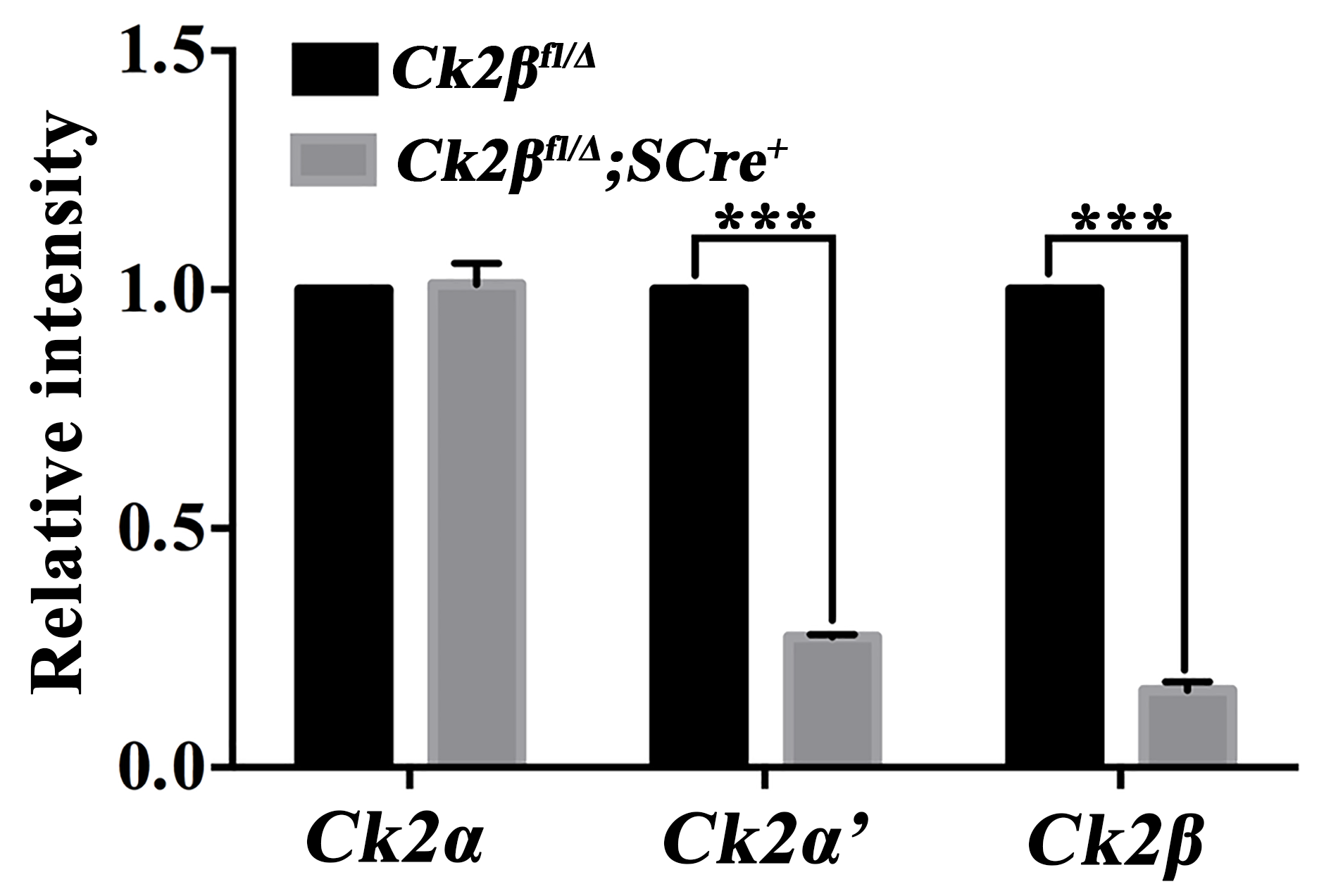

Supplement: Supplementary file 3 [file CPR-53-e12726-s003.png]

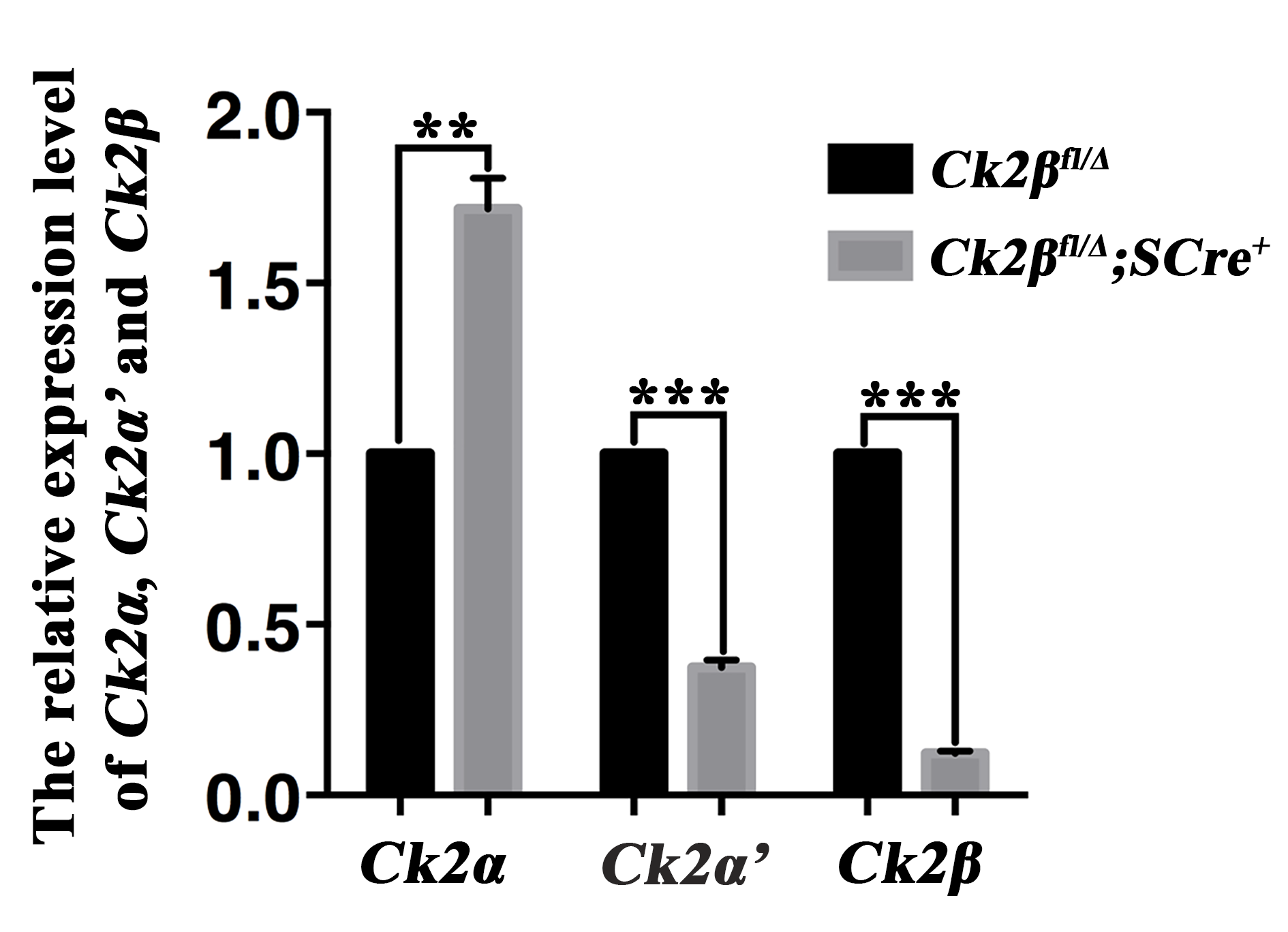

Supplement: Supplementary file 4 [file CPR-53-e12726-s004.png]
